# Long Noncoding Competing Endogenous RNA Networks in Age-Associated Cardiovascular Diseases

**DOI:** 10.3390/ijms20123079

**Published:** 2019-06-24

**Authors:** Simona Greco, Carlo Gaetano, Fabio Martelli

**Affiliations:** 1 Molecular Cardiology Laboratory, IRCCS Policlinico San Donato, San Donato Milanese, 20097 Milan, Italy; 2 Laboratory of Epigenetics, Istituti Clinici Scientifici Maugeri IRCCS, 27100 Pavia, Italy; carlo.gaetano@icsmaugeri.it

**Keywords:** cardiovascular disease, aging, microRNA, long noncoding RNA, competing endogenous RNA

## Abstract

Cardiovascular diseases (CVDs) are the most serious health problem in the world, displaying high rates of morbidity and mortality. One of the main risk factors for CVDs is age. Indeed, several mechanisms are at play during aging, determining the functional decline of the cardiovascular system. Aging cells and tissues are characterized by diminished autophagy, causing the accumulation of damaged proteins and mitochondria, as well as by increased levels of oxidative stress, apoptosis, senescence and inflammation. These processes can induce a rapid deterioration of cellular quality-control systems. However, the molecular mechanisms of age-associated CVDs are only partially known, hampering the development of novel therapeutic strategies. Evidence has emerged indicating that noncoding RNAs (ncRNAs), such as long ncRNAs (lncRNAs) and micro RNAs (miRNAs), are implicated in most patho-physiological mechanisms. Specifically, lncRNAs can bind miRNAs and act as competing endogenous-RNAs (ceRNAs), therefore modulating the levels of the mRNAs targeted by the sponged miRNA. These complex lncRNA/miRNA/mRNA networks, by regulating autophagy, apoptosis, necrosis, senescence and inflammation, play a crucial role in the development of age-dependent CVDs. In this review, the emerging knowledge on lncRNA/miRNA/mRNA networks will be summarized and the way in which they influence age-related CVDs development will be discussed.

## 1. Introduction

Better healthcare and living conditions have contributed to an increase in people’s longevity, which has resulted in a higher prevalence of age-related debilitating and life-threatening diseases such as cardiovascular diseases (CVDs), cancer and neurodegeneration. 

CVDs share with infectious diseases the worst rate of morbidity and mortality, with now more than 30% of all deaths worldwide according to the World Health Organization [1], and it is expected that, as the world population ages, this situation will worsen [2,3]. 

Indeed, age represents a major independent risk factor for cardiovascular-related morbidity and mortality [4,5]. Aging is characterized by a progressive decline in numerous physiological processes, worsening the outcome of other diseases, such as diabetes mellitus, hypertension and coronary disease [6,7,8]. Increased CVD prevalence is also associated with frailty, a condition of increased vulnerability to stressors [9]. In view of this, it is conceivable to define unsuccessful cardiovascular aging a disease per se [10]. Thus, preserving a well-functioning cardiovascular system, which delivers oxygenated blood to all body tissues, is a prerequisite for the good health of all organs and for longevity.

An ever-increasing number of evidence indicates that noncoding RNAs (ncRNAs), such as long ncRNAs (lncRNAs) and micro RNAs (miRNAs) are implicated in most pathophysiological mechanisms. By inhibiting post-transcriptionally both protein coding- (mRNAs) and noncoding-genes, miRNAs regulate gene expression [11,12]. LncRNAs can exert sponging-like effects on both miRNAs and mRNAs, and have both beneficial and detrimental effects. Moreover, the lncRNA family can also regulate molecular processes by acting as host transcripts for miRNAs [13,14].

There are several pieces of evidence reporting that ncRNAs are causally linked to the development of age-associated CVDs by regulating inflammation, cell proliferation, apoptosis, senescence and autophagy. Herein, we will review and summarize the mechanistic, functional, and pathological role of the lncRNAs/miRNAs/mRNA networks in these events and their specific involvement in age-related CVDs.

## 2. Aging and Cardiovascular System Deterioration

Several studies suggest that cardiomyocyte apoptosis and vascular stiffness are associated with aging-induced structural and functional alterations, but the underlying mechanisms are yet to be fully understood. Moreover, the health of the arterial and cardiac systems are mutually correlated because the age-dependent increase of arterial stiffness induces, as compensatory mechanisms, myocardial hypertrophy and fibrosis, which in turn lead to the reduced cardiac output, frequently observed in the elderly. 

Considering these facts, we will here summarize the main age-related pathophysiological processes, both in the vascular system and in the heart.

### 2.1. Vascular Functional Impairment

Endothelial dysfunction and generalized central arterial stiffness are two major vascular modifications caused by aging [7].

Atherosclerosis is a chronic silent inflammatory disease, which can evolve to plaque rupture and thrombosis [15]. The Framingham Heart Study has demonstrated that endothelial dysfunction is independent of clinical disease, as it is also observed in healthy old adults, but creates a pro-atherogenic environment, facilitating the formation of plaques [16]. If other atherosclerotic risk factors are also present (e.g., cigarette smoking, hypertension, high levels of serum cholesterol and its fractions, low levels of HDL and diabetes mellitus), these alterations can lead to the enlargement and rupture of an atherosclerotic stable plaque. 

ECs (Endothelial Cells), VSMCs (Vascular Smooth Muscle Cells), and macrophages are the primary cells contributing to the formation of atherosclerotic lesions. The atherosclerotic plaque results from the infiltration of circulating monocytes in the sub-endothelial space, their differentiation into macrophages, followed by the internalization of modified lipoproteins with transformation in foam cells [17,18].

**Figure 1:**
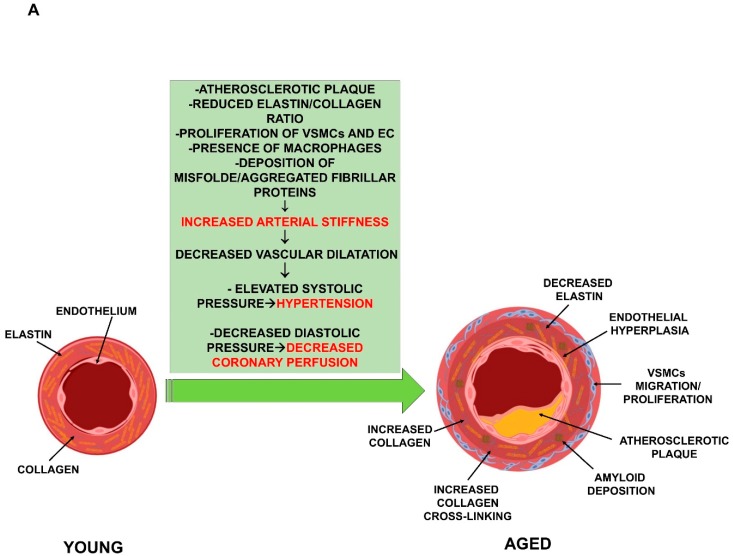

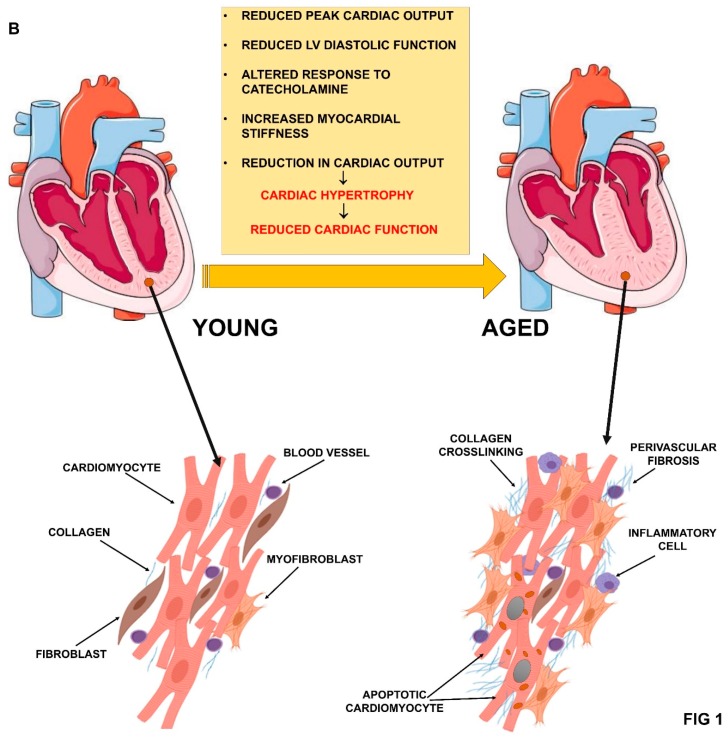
Cardiovascular function deterioration by ageing. (**A**) *Vascular function impairment by ageing.* The loss of aorta distensibility with ageing is associated to: (1) increased collagen deposition; (2) the depletion of elastin; (3) the non-enzymatic glycosylation of collagen, which results in the cross-linking of adjacent proteins; (4) amyloid deposition in the medial layer; (5) migration/proliferation of VSMCs and (6) endothelial hyperplasia. The impaired distensibility is responsible for the development of hypertension and decreased coronary perfusion. (**B**) *Cardiac function impairment by ageing.* The ageing process of the cardiac system is characterized by a reduction of both peak cardiac-output and left ventricular (LV) diastolic function, an altered response to catecholamine, an incomplete relaxation during early diastolic filling, and increased myocardial stiffness. These responses are compensated by increased muscle mass that leads to cardiac hypertrophy and LV wall thickening. This compensatory mechanism enhances cardiac output at the beginning, but reduces the cardiac function as hypertrophy increases. Fibroblast activation to myofibroblast, increased apoptosis, collagen deposition and crosslinking, inflammatory cells migration and perivascular fibrosis are frequent histologic findings in the old myocardium.

Another typical age-related vascular alteration is the increase of arterial stiffness. Many studies have demonstrated, throughout the vascular tree, a gradual, age-related impairment of arterial compliance, diffuse intimal and medial thickening and reduced distensibility of central arteries, which result in decreased vascular dilatation and elevated systolic pressure [19,20]. These changes represent major risk factors for the development of atherosclerosis, hypertension, stroke, and arterial fibrillation [7]. In particular, the loss of aorta distensibility associated with ageing is a very complex process involving several mechanisms. One is the inversion of the elastin/collagen ratio. This is due to both increased collagen deposition and depletion of elastin, mediated by the activation of the matrix enzymes produced by the inflammatory cells in the aorta [21]. Another mechanism is the non-enzymatic glycosylation of collagen present in the arterial wall, which results in the cross-linking of adjacent proteins [21,22]. The fragmentation and calcification of elastic fibers, the increased deposition of collagen and collagen cross-linking, amyloid deposition in the medial layer, and migration/proliferation of VSMC are frequent histologic findings in aged vessels (Figure 1A). In addition, in the elderly, increased levels of markers of both EC oxidative stress and inflammation have been reported [23,24,25]. Initially, the cardiovascular system is able to counteract the stress by moderately increasing the adrenergic signaling. When the stress becomes chronic, however, these responses are strengthened by the activation of the renin-angiotensin-aldosterone and endothelin signaling mechanisms. The activation of these signaling pathways induces an exaggerated chronic inflammatory response and, consequently, more oxidative stress and age-associated structural and functional arterial remodeling [26,27]. Thus, the mechanisms accompanying “physiologic” arterial aging become, with chronic stress, “pathophysiologic”. Moreover, epigenetic control mechanisms are also critically involved in atherosclerosis plaque development and vulnerability [28,29,30].

### 2.2. Cardiac Function Impairment

There is a variety of natural and pathological insults to the myocardium that, throughout life, causes attrition of cardiomyocytes, including ethanol or drugs abuses, increased food intake, viral myocarditis and myocardial infarctions.

The aging process of the cardiac system is characterized by a reduced peak cardiac output and left ventricular (LV) diastolic function, an altered response to catecholamine, an incomplete relaxation during early diastolic filling, and increased myocardial stiffness [10,19]. Moreover, the reduction in cardiac output stimulates a compensation mechanism by increasing muscle mass that leads to cardiac hypertrophy and LV wall thickening [31,32]. This mechanism enhances cardiac output at the beginning, but reduces the cardiac function as hypertrophy increases [33]. In addition, there is an asymmetric growth of the interventricular septum leading to a change in the heart shape [34,35] (Figure 1B).

Increased apoptosis and necrosis are frequent histologic findings in the myocardium of old animals and humans [36,37]. Moreover, cardiomyocytes, as well as other post-mitotic cells, are more likely, during aging, to accumulate the granular pigment ‘lipofuscin’, which is composed of oxidized lipids, cross-linked proteins and oligosaccharides, and is considered as a marker of cellular aging [38].

A typical process associated with ageing is the decline in the number of cardiomyocytes, and this process is more pronounced in males than females [39]. Moreover, the replacement of cardiomyocytes is a very rare event [40,41], due to the withdrawal of cardiomyocytes from the cell cycle after birth [42]. This cell number decline leads to an adaptive hypertrophy of the remaining cardiomyocyte and, eventually, to a reduction of cardiac capacity [31,32].

A decrease in autophagy efficiency has been found to be associated with the ageing process, determining the abnormal deposition of intracellular protein aggregates, enhanced ROS production, decreased ATP production, and cell death [43,44]. 

Several neurodegenerative diseases, such as Alzheimer’s, Huntington’s and Parkinson’s diseases, are characterized by amyloidosis, which is the accumulation of misfolded proteins as intracellular or extracellular aggregates. Some cardiomyopathies share these features with neurodegenerative diseases. Recent evidence indicates that not only the accumulation of β-amyloid fibrils in extracellular plaques, but also the soluble intermediates of fibril formation seem to be toxic [45]. Indeed, soluble oligomers have been found in animal models of HF as well as in human HF [46,47,48,49,50,51]. Another amyloid-protein, the transthyretin (TTR), is a tetrameric protein synthesized mostly by the liver. This protein can be deposited as insoluble fibrils into the heart as consequence of protein misfolding due to gene mutations or as an ageing-related phenomenon, such as the age-dependent increased oxidative stress [52,53,54]. The pathogenitic role of senile amyloidosis in dilated cardiomyopathies is also demonstrated by the accumulation of wt TTR in patients with heart failure with preserved ejection fraction (HFpEF) [55,56].

## 3. The Noncoding RNAs Network

### 3.1. microRNAs

miRNAs are small non-coding RNA sequences, ~22 nucleotide-long, that, by partially interacting with target mRNAs, lower their translation and/or stability [12]. The primary miRNA transcript, the pri-miRNA, is longer than the mature form and, after cleavage by the microprocessor complex (containing the ribonuclease Drosha), generates the miRNA precursor (pre-miRNA) that is exported to the cytoplasm [57]. After translocation to the cytoplasm, the pre-miRNA is cleaved by Dicer to generate a ~22-bp-long duplex RNA. Only one strand of the duplex represents the mature miRNA, and it is loaded onto the *RNA-induced silencing complex* (*RISC*), which contains Argonaute (Ago) proteins [58]. This complex can target a family of target mRNAs by partial hybridization, frequently at the miRNA ‘seed’ region (nucleotides 2–7). In this way, in most circumstances, this complex acts as a negative regulator of gene expression. Worth noting is that more than one miRNAs can recognize the same mRNA generating a complex miRNA/mRNA network. miRNAs mediate the regulation of gene expression in virtually all aspects of cell biology, and dysregulation of miRNAs has been causally linked to several age-dependent CVDs [13].

### 3.2. Long Noncoding RNAs

The threshold of 200 nucleotides usually separates short from long ncRNAs. The biogenesis and turnover of most lncRNA are closely similar to those of the mRNAs coding for proteins. As for mRNAs, lncRNA transcription is mediated by promoter elements, transcription factors and histone modifications [59].

According to their genomic localization and biogenesis, lncRNAs can be classified as: (1) intergenic lncRNA (lincRNA), transcribed independently of protein-coding genes; (2) antisense lncRNAs (lncRNA-AS), when they are expressed from the opposite strand of mRNAs; (3) pseudogene-encoded lncRNAs, that are transcribed from vestigial genes that lost their protein-coding potential; (4) intronic lncRNAs, when they are present in introns of coding genes; (5) promoter-associated lncRNAs, if they are transcribed from the promoter regions of coding mRNAs [13,60].

**Figure 2:**
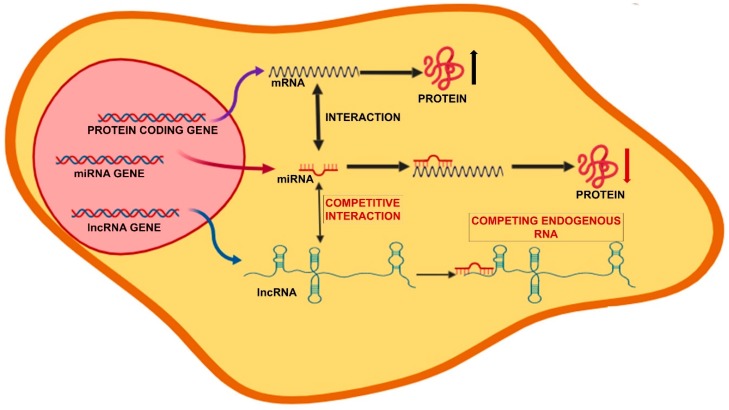
Competitive interactions between lncRNAs, miRNAs and mRNAs. The presence of miRNA response elements (MREs) allows the binding of miRNAs, which results in the inhibition of mRNA expression and/or stability. In addition, lncRNAs acting as ceRNAs harbor MREs and function as “sponges” for the miRNAs, which are sequestered so that the miRNA-RISC is unable to regulate the expression of target mRNAs. Thus, by acting as ceRNAs, lncRNAs are competitors for the binding of miRNA to the mRNA targets.

The lncRNA can function through different mechanisms of action: (1) Epigenetic regulation, when they act as scaffolds, bridges, and tethers of factors that regulate the state of the chromatin; (2) Transcriptional regulation, when they modulate the rates of RNA polymerase II initiation and elongation; (3) Nuclear compartmentalization, to maintain nuclear structures; (4) Post-transcriptional gene regulation by both basepairing with mRNAs, or acting as cofactors or competitors of RNA-binding proteins; (5) Competing endogenous RNAs (ceRNAs), when lncRNAs can function as decoys or sponges for miRNAs [61,62]. In particular, lncRNAs acting as ceRNAs harbor miRNA response elements for binding miRNAs and generally display increased expression and/or stability compared to other lncRNAs. The presence of one or multiple binding site and the high level of the ceRNA make it able to sequester the target miRNA, thus titrating the miRNA-RISC away from the mRNAs it regulates [63,64]. Thus, by acting as ceRNAs, lncRNAs are at the center of a large-scale regulatory network across the transcriptome, greatly expanding the complexity of gene expression regulation (Figure 2).

There are clearly several complications of the system and miRNA sponging may become biologically relevant only for a small subset of ceRNAs/miRNAs whose cellular concentration and target abundance meet a narrow range of values [65]. Other relevant factors are the affinity of the competitive binding to the miRNA for the lncRNA and the mRNA, as well as the miRNA ability to induce the degradation of the bound RNA (both lncRNA and mRNA). It should also be considered that more than one miRNA has multiple target mRNAs and possibly more than one ceRNA-lncRNA may be part of the same network, yielding an extremely complicated system that may provide stability to the structure. 

In spite of all these complications and limitations, an ever increasing number of miRNA/lncRNA/mRNA networks are emerging as regulators of a variety of age-associated conditions [11,66,67].

## 4. miRNA/lncRNA/mRNA Network Modulation by Age-Related Mechanisms

The altered signaling observed during the aging of the cardiovascular system involves several mechanisms, such as a reduced response to acute stress, reduced autophagy, higher levels of markers of chronic stress, including reactive oxygen species (ROS) and inflammation, increased cell death and necrosis, with reduced cell replacement [68,69]. 

These cellular events, in general, have detrimental consequences and eventually can determine the development of age-associated pathologies. As an example, the accumulation of ROS sensitizes the heart to the renin-angiotensin-aldosterone system, inducing apoptosis and increasing the propensity for adverse cardiac remodeling, diastolic dysfunction and heart failure [70].

In the following paragraphs, we will review the role of autophagy, inflammation and senescence in the modulation of the lncRNA/miRNA/mRNA networks and their involvement in age-dependent CVDs (summarized in Table 1).

### 4.1. Autophagy Impairment 

Autophagy is a fundamental process regulating cellular quality control. The term “autophagy” means “self-eating” in Greek, and it is an intracytoplasmic degradation process of organelles such as mitochondria, endoplasmic reticulum and peroxisomes, as well as intracellular pathogens [92]. Autophagy leads to the dynamic recycling of protein aggregates, thus providing both energy and building material for new protein and membrane production [70]. In particular, the autophagy related protein (ATG) complexes coordinate protein degradation by the formation of the autophagosome, which, in turn, fuses with lysosomes to generate autolysosome. Inflammation, oxidized lipoprotein, ER stress and ROS production are some of the factors that induce the autophagic process [93]. A decrease in the efficiency of autophagy determines the abnormal deposition of intracellular protein aggregates, enhanced ROS production, decreased ATP production, and cell death [43,44]. 

Several data demonstrate that physiological autophagy is a protective mechanism that serves to maintain normal cardiovascular function [70]. Actually, impaired autophagy is associated with CVDs development [68,69]. 

In particular, autophagy, by degrading the damaged intracellular organelles, promotes the survival of the cellular components of the plaque and reduces the accumulation of ROS [68]. This activity stabilizes the plaque, thus preventing its rupture and the consequent detrimental effects, such as arterial occlusion, acute coronary syndrome, myocardial infarction, and stroke [69]. Moreover, autophagy, by degrading the accumulated dysregulated proteins, decreases the cardiac mass, counteracting ventricular hypertrophy [94]. Autophagy is also induced in ischemia and reperfusion (I/R) [95]. Indeed, during I/R, the oxygen supply limitation to the heart activates the autophagic process as a homeostatic mechanism to protect the myocardium from further ischemia [96].

It is noteworthy that, in spite of its well-known cardiovascular protective effects, dysregulated autophagy can also have detrimental effects. Actually, pressure overload triggers autophagy, which in turn leads to cell death, thus worsening heart failure, and plaque destabilization [69]. 

Autophagy can play a negative role also in I/R. In fact, following I/R injury, a burst of oxidative stress occurs, which results in enhanced cardiomyocyte autophagy followed by cell apoptosis [97].

**Figure 3:**
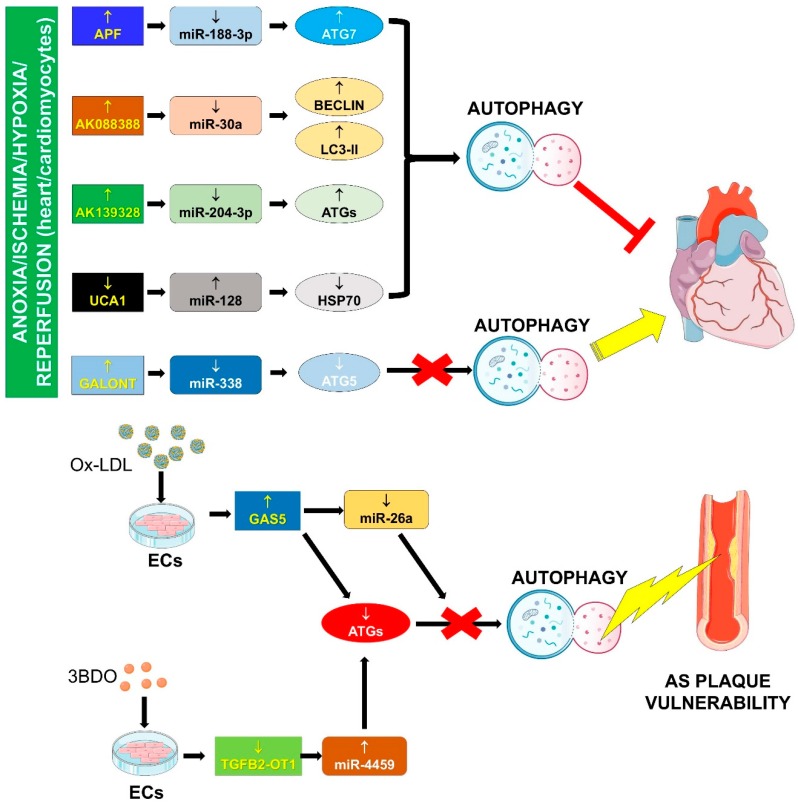
LncRNA-miRNA-mRNA networks and autophagy. A decrease in autophagic function is a hallmark of ageing. A variety of stimuli, such as hypoxia or ischemia/reperfusion, oxidized lipoproteins (ox-LDL) or treatment with an MTOR pathway agonist (3BDO), can activate the interaction of lncRNA and miRNA, and the specific modulation of direct and/or indirect targets that, in turn, can affect positively or negatively the autophagic process.

The factors determining whether autophagy will be adaptive or detrimental are still unknown, but the extent and duration of autophagy seem to be important [98]. The involvement of the lncRNA/miRNA/mRNA network in autophagy is reported in the following sub-paragraphs and summarized in Figure 3. 

#### 4.1.1. APF/miR-188-3p/ATG7

Wang and collaborators investigated the role of the network APF/miR-188-3p/ATG7 in the regulation of the I/R-induced autophagy [71]. In particular, the expression of the lncRNA APF (also named AH079427) increases in cardiomyocytes treated with anoxia/reperfusion, as well as in vivo after I/R [71]. The exogenous expression of APF promotes both autophagy and cell death and these effects are mediated by sequestration of miR-188-3p and induction of the pro-autophagic gene ATG7. Accordingly, in vivo, the silencing of APF leads to decreased autophagy and reduction of myocardial infarction size and thus to an amelioration of myocardial functions.

#### 4.1.2. AK088388/miR-30a/Beclin-1 and LC3-II

LncRNA AK088388 has been recently found to modulate autophagy in cardiomyocytes functioning as an endogenous RNA sponge of miR-30a under Hypoxia/Reperfusion conditions [72]. The inhibition of AK088388 is linked to increased levels of miR-30a and attenuation of the expression of Beclin-1 and LC3- II. This effect leads to a reduction in cardiomyocyte damage and autophagy [72].

#### 4.1.3. AK139328/miR-204-3p/ATGs

AK139328 is another lncRNA induced by I/R alone or in combination with diabetes (DM/IR) [73]. Interestingly, the inhibition in vivo of AK139328 in DM/IR reduces LV enlargement, fibrosis and myocardial infarct size, improving the heart function [73]. Accordingly, AK139328 silencing in DM/IR derived cardiomyocytes attenuates autophagy and apoptosis [73]. Mechanistically, AK139328 sponges miR-204, an anti-autophagy miRNA also important for metabolic recovery after I/R [73]. Indeed, the simultaneous silencing of AK139328 and overexpression of miR-204 relieves the hypoxia/reperfusion injury through inhibition of the autophagic cascade [73]. 

#### 4.1.4. BACE1-AS/miRNAs/BACE1

The abnormal deposition of intracellular protein aggregates such as amyloid has been found to be associated with a decreased efficiency in autophagy with ageing [43,44]. Recently, the overexpression of the lncRNA beta-secretase 1-antisense RNA (BACE1-AS) in post-ischemic HF has been related to both the accumulation of BACE1 mRNA, which encodes for the enzyme responsible for β–amyloid, and the cardiac deposition of β–amyloid [51]. Indeed, the dysregulation of the network formed by BACE1-AS, BACE1 and β–amyloid has been related to the cell toxic effect of β–amyloid [51]. Different miRNA-mediated mechanisms have been proposed through which BACE1-AS mediates the stabilization of BACE1 mRNA. Indeed, BACE1-AS can act by masking the binding site for miR-485-5p on BACE1 mRNA [74]. Moreover, BACE1-AS shares with BACE1 the miRNA-responsive-elements for miR-29, miR-107, miR-124, miR-485 and miR-761, acting as a ceRNA preventing BACE1 targeting [75]. 

#### 4.1.5. Galont/miR-338/ATG5

“GATA1 activated lncRNA” or Galont is induced by anoxia/reperfusion in neonatal cardiomyocytes [76], and its overexpression triggers both autophagic and apoptotic responses. miR-338 is a Galont direct target and its expression is repressed in anoxia/reperfusion [76]. Indeed, Galont overexpression in vitro reduces both autophagy and apoptosis induced by anoxia/reperfusion through the inhibition of miR-338 target ATG5 [76]. 

#### 4.1.6. GAS5/miR-26a/ATGs 

GAS5 is an lncRNA that has been found to be increased in atherosclerosis and its silencing reduces the apoptosis of macrophages and ECs after treatment with oxidized low density lipoproteins (ox-LDL) [99]. Very recently, Liang et al. [77] investigated the mechanisms underpinning these effects. The expression of GAS5 and miR-26a, which interacts with the lncRNA, are up- and down-regulated, respectively, in both plasma samples from patients suffering from aortic stenosis and in aortic ECs treated with ox-LDL. The treatment of ECs with ox-LDL determines the increase of cell apoptosis and the decrease of autophagy. GAS5 silencing and treatment with ox-LDL leads to increased expression of miR-26a and of autophagic markers [77]. These data suggest a novel regulatory mechanism for ox-LDL-induced impaired autophagy flux in ECs, with GAS5/miR-26a axis that might provide potential targets in the process of atherosclerosis.

#### 4.1.7. UCA1/miR-128/HSP70

In both mouse cardiomyocyte H9c2 cells under Hypoxia/Reperfusion or rat hearts undergoing I/R, the expression of miR-128 has been found to be increased, while the level of its target heat shock protein 70 (HSP70) was decreased [79]. The post-conditioning by morphine treatment leads to the increase of lncRNA urothelial carcinoma-associated 1 (UCA1) level, which, by sponging miR-128, results in the increase of HSP70 and the attenuation of cell autophagy and cardiac injury [79].

#### 4.1.8. TGFB2-OT1/miR-4459/ATG13

The lncRNA TGFB2-OT1 (TGFβ2 overlapping transcript 1 or FLJ11812) is located in the 3′UTR of TGFβ2 (transforming growth factor, β2). Ge et al. reported that TGFB2-OT1 can sequester miR-4459, thus increasing the level of its target ATG13 (autophagy related 13) and promoting autophagy [78]. In particular, the treatment of ECs with the small molecule 3BDO (3-benzyl-5-((2-nitrophenoxy) methyl)–dihydrofuran-2(3H)-one) inhibits both rapamycin-induced autophagy and the expression of TGFB2-OT1 [78], and activates the MTOR signaling [100]. MTOR activation phosphorylates TIA1 (TIA1 cytotoxic granule-associated RNA binding protein), which, in this state, represses the expression of TGFB2-OT1 [71]. The reduced expression of TGFB2-OT1, which competes with miR-4459 for binding to ATG13, leads to an inhibition of autophagy [78]. Thus, the modulation of TGFB2-OT1/miR-4459/ATG13 axis could represent a potential therapeutic approach for autophagy regulation in CVDs.

### 4.2. “Inflammageing” 

An hallmark of ageing is also represented by the presence, even in the absence of risk factors and clinically active diseases, of high levels of pro-inflammatory markers in cells and tissues of older individuals, a condition that is called “inflammageing” [101,102].

It is not yet known whether high levels of tissue and circulating pro-inflammatory molecules might contribute to associated pathological conditions or whether they represent only reactive markers of the underlying pathology. Nevertheless, clear evidence indicates that inflammageing is a risk factor for CVDs [103,104].

Inflammation plays a crucial role in fighting infections or extraneous molecules, but, despite this protective role, sustained and prolonged inflammation might be detrimental to health. Actually, during inflammation, ECs are affected by an early damage, which, in turn, might be pro-atherogenetic. Atherosclerotic plaques can in turn produce additional pro-inflammatory molecules, amplifying the role of inflammation in atherogenesis [69,105,106].

Noncoding RNAs, including miRNAs and lncRNAs are emerging regulators of the inflammatory signalling. Several ncRNAs have been so far identified to be involved in the regulation of NF-κB signalling and inflammation [107,108,109]. 

Moreover, another layer of complexity added to vascular ageing is represented by the link between oxidative stress and inflammation, both leading to endothelial dysfunction [110]. Indeed, an increase in inflammatory cytokines and chemokines leads to infiltration of T cells and macrophages, but also to the internalization of oxidized lipoproteins (ox-LDL) with transformation in foam cells, both contributing to tissue injury [17,18]. 

**Figure 4:**
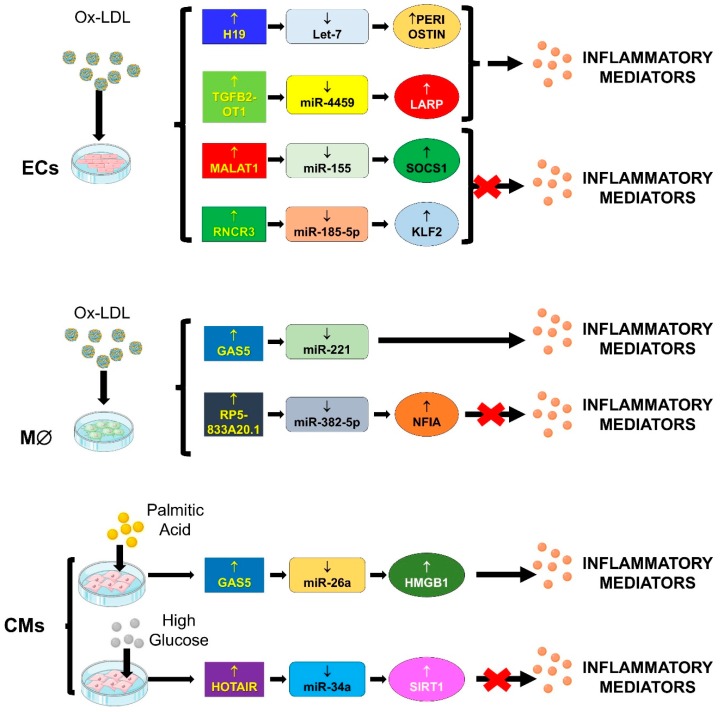
LncRNA-miRNA-mRNA networks and inflammation. During ageing, there is an increased accumulation of inflammatory molecules as well as a burst in oxidative stress. A prolonged state of inflammation can be detrimental for cardiovascular tissue and it can predispose to atherogenesis. Indeed, an increase in inflammatory cytokines and chemokines leads to the internalization of oxidized lipoproteins (ox-LDL) with transformation in foam cells, both contributing to tissue injury. The figure summarizes in vitro experiments performed in endothelial cells (ECs), macrophages cells (M∅) and cardiomyocytes (CMs).

Thus, the role of lncRNA/miRNA/mRNA network in cardiovascular physio-pathology will be discussed in light of these processes and findings will be summarized in Figure 4.

#### 4.2.1. TGFB2-OT1/miR-4459, miR-3960 and miR-4488/CERS1, NAT8L, ATG13 and LARP

Autophagy and inflammation may play a coordinated role in the progression of cardiovascular events [111,112]. In particular, autophagy regulates the inflammation cascade by both clearing mitochondrial ROS and inhibiting NF-kB activation, thus preventing further injury to the atherosclerotic plaque [113,114]. In the previous section, the role of TGFB2-OT1/miRNAs network in autophagy has been discussed [78]. Works from the same research group that identified this function has showed that TGFB2-OT1/miRNA interaction plays a role in inflammation too [87]. Indeed, Huang and colleagues [87] have shown that the expression of TGFB2-OT1 is induced in ECs treated with LPS (lipopolysaccharide) or ox-LDL and that this increase is mediated by NUPR1 which, in turn, induces TIA1, responsible for TGFB2-OT1 processing from 3′UTR of TGFB2 [78]. TGFB2-OT1 acts as a ceRNA of miR-4459 [78], as well as of miR-3960 and miR-4488 [87]. The sponging of these miRNAs, in turn, augments the protein levels of their targets CERS1 (ceramide synthase 1), NAT8L (N-acetyltransferase 8-like [GCN5-related, putative]), ATG13 and LARP1 (La ribonucleoprotein domain family, member 1). LARP1 further increases the levels of SQSTM1 (Sequestosome 1), ATG3 and ATG7. Worth noting is that CERS1 and NAT8L regulate autophagy by modulating mitochondrial function. ATG3, ATG7 and ATG13 also regulate autophagy, while increased SQSTM1 levels activate RELA and CASP1, inducing the release of the inflammatory cytokines. Collectively, these data indicate that ECs autophagy and inflammation are both promoted by TGFB2-OT1 expression.

#### 4.2.2. GAS5/miR-26a/HMGB1

The saturated fatty acid palmitic acid (PA) induces an inflammatory phenotype in cardiomyocytes, characterized by increased release of pro-inflammatory cytokines and oxidants, leading to cellular hypertrophy and apoptosis [115,116]. The expression of GAS5 is increased in PA-treated mouse cardiomyocytes (H9c2 cells), and silencing in vitro experiments have demonstrated that GAS5 is involved in both the release of inflammatory mediators and cellular injury [80]. In vitro assays have validated the bioinformatics prediction of GAS5 as interactor and of high mobility group box 1 (HMGB1) as target of miR-26a, suggesting that the network GAS5/miR-26a/HMGB1 may have a role in the mechanism of myocardial lipotoxic injury [80]. 

#### 4.2.3. GAS5/miR-221/MMPs 

In ox-LDL-stimulated macrophages, the expression of GAS5 is increased and its elevation aggravates ox-LDL-induced inflammation by inducing IL-6, TNF-α and IL-1β, while its silencing reversed ox-LDL-induced inflammation [81]. GAS5 expression is also increased in atherosclerotic plaques of aortic stenosis patients [81]. The pro-inflammatory effects of GAS5 on ox-LDL-stimulated macrophages are mediated by the sponging miR-221 and the consequent elevation of MMP-2 and MMP-9. 

#### 4.2.4. H19/let-7/PERIOSTIN

Cao et al., [82] have showed that H19 expression is increased in the serum of patients with atherosclerosis and in the ox-LDL-treated human umbilical vein endothelial cells (HUVECs). The effects of H19 silencing reverses the ox-LDL-mediated cell toxicity, and the secretion of inflammatory mediators and reactive oxygen species (ROS). The inflammatory and oxidative roles of H19 are mediated by periostin overexpression due to H19 sponging of let-7a [82].

#### 4.2.5. HOTAIR/miR-34a/SIRT1

Very recently, the importance of HOTAIR has been evaluated in H9c2 rat cardiomyocytes exposed to high glucose (HG) and in a mouse model of experimental diabetes, showing that HOTAIR functions as a ceRNA to upregulate SIRT1 by sponging miR-34a [83].

HOTAIR is a lncRNA located at the antisense strand of the HOXC gene locus [117], and its modulation has been identified to be associated with CVDs [118,119,120]. 

In cardiomyocytes treated with HG, the silencing of HOTAIR induces a higher expression of inflammatory cytokines, ROS and apoptosis compared to cells treated with HG only [83]. Moreover, in diabetic mice, the cardiac tissue infiltration of inflammatory cells was reduced by HOTAIR overexpression, indicating an anti-inflammatory role of HOTAIR [83]. HOTAIR silencing de-represses miR-34a, reducing the expression of miR-34a target SIRT1 [83], a well-known anti-ageing factor, repressor of inflammation and oxidative-stress [121,122]. Indeed, in diabetic mice, SIRT1 knockout increased the leukocytes infiltration, the oxidative stress and, thus, the cardiac performance [83].

#### 4.2.6. MALAT-1/miR-155/SOCS1

Several evidence indicates that the lncRNA MALAT-1 plays a vascular protective role. In particular: (1) MALAT1 regulates the migration of vascular ECs as well as vascular growth, both in vitro and *in vivo*, (2) it protects ECs against ox-LDL-induced dysfunction and (3) its expression is upregulated in response to high glucose treatment [123,124,125]. The expression of this lncRNA has been found to be increased in human coronary artery ECs upon ox-LDL stimulation. The ox-LDL treatment of the ECs in combination with MALAT-1 silencing stimulates the release of inflammatory factors, while MALAT-1 overexpression reduces the release of cytokines and apoptosis [84]. Interactor of MALAT-1 is miR-155, which, in turn, is induced by ox-LDL [126], and a relevant target of miR-155 is SOCS1, involved in atherosclerosis inflammation [127]. Thus, the activation of the pathway MALAT-1/miR-155/SOCS1 by repressing JAK/STAT signaling and reducing the release of cytokines, relieves the inflammation of ECs mediated by ox-LDL [84].

#### 4.2.7. RNCR3/miR-185-5p/KLF-2

The lncRNA RNCR3 has a protective effect against inflammation in the atherosclerotic lesions, and this function is mediated by a crosstalk between KLF2 and RNCR3 through the interaction with miR-185-5p [85].

Indeed, RNCR3 has been found to be upregulated in human and mouse atherosclerotic plaques; its knock-down in ApoE^−/−^ mice and after high-fat diet increases atherosclerosis, cholesterol and triglycerides plasma levels and the release of inflammatory mediators, indicating an anti-atherosclerotic role of RNCR3 [85]. Mechanistically, RNCR3, by sponging miR-185- 5p, induces the expression of its target KLF-2, a transcriptional factor conferring an endothelial vasoprotective phenotype [128]. Of interest is the fact that the atherosclerotic segments of aortas from apoE^−/−^ mice expressed higher levels of both RNCR3 and KLF2. Similar data were obtained comparing human aortic atherosclerotic plaques with surrounding normal aortic tissue, confirming the relevance of the mouse model.

Thus, in the atherosclerotic plaque, the upregulation of the RNCR3/KLF-2 axis, by reducing inflammation, lipid accumulation and atherosclerosis extent, might be protective against CVDs.

#### 4.2.8. RP5-833A20.1/miR-382/NFIA

The lncRNA RP5-833A20.1 is located in the second intron of the Nuclear Factor IA (NFIA) sequence, and its transcription direction is opposite to that of NFIA. Microarray analysis of human macrophage–derived foam cells shows that RP5-833A20.1 is upregulated, while NFIA expression is downregulated [86]. A similar modulation has been demonstrated when macrophages are exposed to both oxidized and acetylated low-density lipoprotein (ox-/Ac-LDL). The down-regulation of NFIA is RP5-833A20.1-dependent and this effect is mediated by the sponging of miR-382-5p, which targets NFIA. Moreover, the network RP5-833A20.1/miR-382/NFIA is also involved in the regulation of lipid accumulation and inflammation. Accordingly, in vivo over-expression of NFIA in apoE^−/−^ mice reduces the atherosclerotic plaque formation [86]. Therefore, NFIA might be considered as a potential target to treat atherosclerotic vascular disease.

### 4.3. Cellular Senescence

There is a complex and not yet fully understood connection between oxidative stress and cell senescence. Senescence was originally observed as a mechanism of permanent withdrawal from the cell cycle after a reproducible number of cell divisions [129]. Senescence is a mechanism induced by both endogenous and exogenous stimuli, which mediate replicative senescence or stress-induced premature senescence (SIPS), respectively [130,131]. The best characterized endogenous stimulus is telomere erosion. Telomeres consist of tandem repeats of the TTAGGG sequence and are involved in the maintenance of genomic and cellular stability and replication [132]. Several mechanisms can shorten the telomere, such as cell division, aging, and ROS accumulation [131,133]. Consequently, cardiovascular risk factors such as smoking, high cholesterol levels and obesity can promote telomere attrition [134]. Among the exogenous stimuli inducing SIPS, oxidative stress is a prominent one. 

Two distinct and partially intersecting pathways can mediate replicative senescence and SIPS. Among the pathways activated by senescence are the p53/p21 (replicative senescence) and the p16Ink4a/retinoblastoma (SIPS) protein pathways, both contributing to cell growth arrest [135,136]. 

Senescence is associated with the senescence-associated secretory phenotype (SASP), which consists in the release of a large number of pro-inflammatory cytokines and growth factors. These factors cause an increased burden of tissue inflammation and oxidative stress that shorten further the telomeres, thus aggravating the senescence process [137]. It is fair to assume that telomere shortening and cellular senescence are involved in aging and in the development of CVDs. Indeed, experiments conducted in the INK-ATTAC mouse model show that elimination of senescent cells can delay ageing-associated disorders [138].

**Figure 5:**
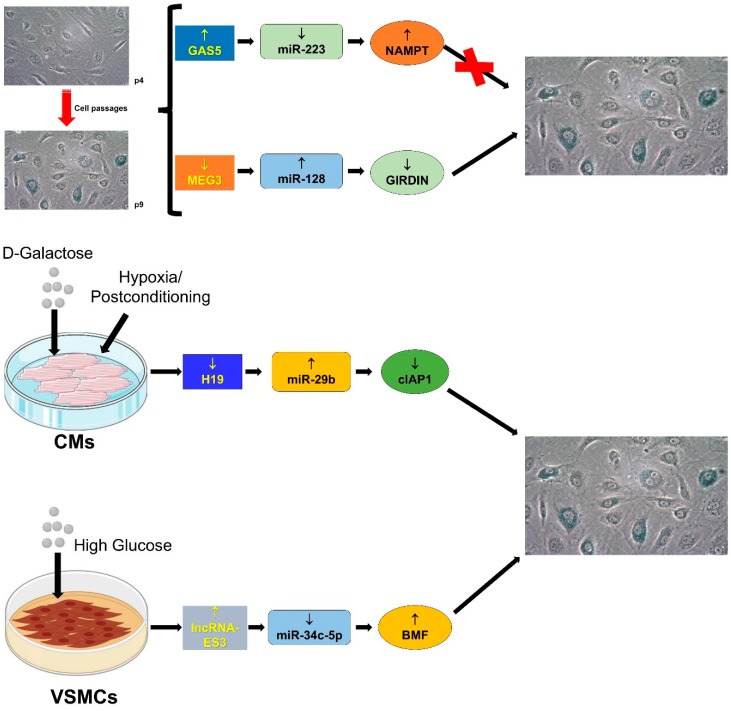
LncRNA-miRNA-mRNA networks and senescence. Cellular senescence is associated with the release of a large number of pro-inflammatory cytokines and growth factors. These factors cause an increased burden of tissue inflammation and oxidative stress that contribute to telomere shortening. Moreover, telomere shortening and cellular senescence are involved in aging and in the development of CVDs. The figure shows that the senescence phenotype can be induced by: (1) ageing the endothelial cells by culturing until late passages (p9) (2) stimulating cardiomyocytes (CMs) with D-galactose and (3) treating vascular smooth muscle cells (VSMCs) with high glucose. The lncRNA-miRNA-mRNA networks triggered by these conditions stimulate or inhibit senescence according to the modulated targets.

There are several ncRNAs involved in the senescence phenotype at transcriptional, post-transcriptional, and post-translational levels (for detailed reviews see: [139,140]). Hereafter, the involvement of the lncRNA/miRNA/mRNA network in senescence is discussed and data summarized in Figure 5.

#### 4.3.1. GAS5/miR-223/NAMPT

Recently, the network GAS5/miR-223/nicotinamide phosphoribosyltransferase (NAMPT) has been associated to the modulation of cellular senescence of endothelial progenitor cells (EPCs) [88]. The lncRNA GAS5, by sponging miR-223 in EPCs at late passages, downregulates miR-223 and subsequently de-represses the expression of miR-223-target NAMPT, which induces cell proliferation and inhibits cellular senescence by acting on PI3K/AKT signaling [88]. 

#### 4.3.2. H19/miR-29b-3p/cIAP1

H19 has been demonstrated to relieve the hypoxia/post-conditioning (H/Post)-associated injury in cardiac cells through miR-29b-3p and its target cIAP1 (Cellular Inhibitor of Apoptosis Protein 1) [89]. D-Galactose derives from the digestion of lactose and, being a reducing sugar, reacts with free amines of amino acids in proteins forming advanced glycation products. In this way, the oversupply of D-galactose could generate advanced glycation products that induce oxidative-stress [141]. Zhang and colleagues have shown that, in neonatal cardiomyocytes, the induction of senescence by D-Galactose reduces H19 levels [89]. Moreover, during H/Post, both the expression of H19 and the cell number increase, but not in senescence cells. Mechanistically, during H/post, the sponging of miR-29b-3p and the increase of miR-29-3p target cIAP1 mediate the anti-apoptotic effect of H19 [89]. 

The ischemic post-conditioning is, normally, a cardio-protective mechanism since it reduces I/R injury [142,143]. Data from Zhang and colleagues, suggest that, in the aged heart, this protective effect can be lost, since the senescence process lowers the levels of H19, decreasing the antiapoptotic potential of post-conditioning [89].

#### 4.3.3. lncRNA-ES3/miR-34c-5p/BMF 

Vascular calcification is a well-known major risk factor for the development of cardiovascular diseases [144,145] and this process increases with ageing. While the underpinning mechanisms that are responsible for calcification still remain elusive, it has been shown that the osteoblastic program is activated in senescent VSMCs [145]. Recently, Lin et al., [90], have found that the expression of miR-34c-5p is reduced in human aorta VSMCs with the senescence/calcification phenotype induced by the hyperglycemic stimulus. Moreover, the overexpression of miR-34c-5p reduces the level of the senescence markers p16 and p21, suggesting that this miRNA is important in counteracting the senescence phenotype [90]. The authors have also demonstrated that the lncRNA-ES3 (LIN00458) is induced in VSMCs cultured in high-glucose and that lncRNA-ES3 interacts with miR-34c-5p repressing its expression and increasing the expression of miR-34c-target BMF (Bcl-2 modifying factor). Both lncRNA-ES3 and BMF overexpression induce the senescence/calcification phenotype, suggesting a role for the lncRNA-ES3/miR-34c-5p/BMF axis in vascular ageing.

#### 4.3.4. MEG3/miR-128/Girdin

MEG3 is a well characterized lncRNA which controls vascularization and angiogenesis, EC proliferation, and senescence [146,147,148]. Girdin, also known as GIV, is involved in the signaling of G protein-coupled receptors, such as VEGF-R [149]. In particular, Lan and colleagues found that both MEG3 and Girdin are downregulated in vessels derived from aged humans or mice [91]. Moreover, reduced levels of MEG3 and Girdin have been found in senescent ECs [91]. By contrast, miR-128, which is associated with senescence [150], displayes an opposite pattern of expression [91]. In keeping with bioinformatics prediction, luciferase assay and RNA pulldown experiments indicate that there is an interplay between MEG3, miR-128 and Girdin, where decreased expression of MEG3 decreases the sponging of miR-128, which can reduce the expression of Girdin [91]. In particular, MEG3 silencing in ECs reduces platelets phagocytosis, membrane fluidity and increases lipoprotein oxidation, ROS accumulation and telomere shortening [91]. Altogether, these effects are characteristics of endothelial senescence and are associated with the onset of the atherosclerotic process.

## 5. Conclusions and Future Perspectives

CeRNA networks are very complex and are influenced by a variety of parameters. As described in the previous sections, the affinity of the miRNA binding site, the ability of the miRNA to induce the degradation of the bound RNA, the relative abundance of direct players are some of the elements that contribute to the complexity of lncRNA/miRNA/mRNA networks, determining the biological outcome.

In spite of all these complications, evidence of the existence and biological relevance of these networks is now clear in virtually all physiological and physio-pathological situations.

In this review, we described the lncRNAs harboring miRNA binding sites and functioning as molecular decoys or sponges by sequestering miRNAs away from other transcripts. However, other types of ncRNA interaction are also relevant for age-related CVDs. Indeed, miRNA sequences may be embedded into lncRNAs that, in this way, can act as progenitors or reservoirs of miRNAs [14,151]. An example is H19, involved in several CVDs and deregulated under cell stress conditions [119,148,152,153], which is the precursor of miR-675 [154]. In particular, the lncRNA H19 via miR-675 regulates the expression of miR-675 target VDAC1 [155]. This pathway modulates mitochondrial apoptosis induced by high-glucose and attenuates hyperglycemia-mediated oxidative stress in myocardial tissue [155]. Another target of miR-675, USP10, has been found to be involved in the modulation of the anti-senescence actions of melatonin on H_2_O_2_-treated C-kit+ cardiac progenitor cells (CPCs) [156].

A particular class of ncRNAs acting as ceRNAs is constituted by circular RNAs (circRNAs), covalently closed RNA circles [157,158]. The prototype is constituted by ciRS-7/CDR1as, expressed mostly in the brain, serving as miR-7 sponge and containing more than 70 binding sites [63,159]. Likewise, *sry*, a testis-specific circRNA, harbors 16 binding sites for miR-138 [159]. While these seem to be extreme cases, many circRNAs harboring only one or two bindings sites have been reported to act as miRNA decoys or sponges, also in the cardiovascular system [157]. It is expected that circRNA ceRNAs may play a role in autophagy, apoptosis, necrosis, senescence and inflammation regulation in CVDs as well.

In view of the fact that both miRNAs and lncRNAs influence mRNA function in CVDs, a clear understanding of how lncRNAs and miRNA interact may be instrumental for developing innovative therapeutic strategies. Currently, significant effort has been made to develop techniques for in vitro and in vivo manipulation of miRNA and lncRNA levels and also to translate these results to a clinical scenario. 

In this respect, one element seems to be of great importance: while miRNAs are in most cases highly conserved, typically, lncRNAs display poor conservation across species, showing conserved “patterns” of bases surrounded by large less conserved sequences [14,160]. This could represent a significant hurdle to the transferability of findings in animal models of disease to humans, since some of the lncRNAs/miRNA networks are based on non-conserved lncRNA sequences. 

Another important element is lncRNA annotation. While greatly improved, the process of lncRNA annotation is far from being completed and much work is still needed before an annotation accuracy compared to that of coding RNAs is obtained. 

In spite of all these difficulties, many lncRNAs/miRNA/mRNA interactions have been identified, and the field is well poised to reveal more insights into how these networks are regulated and function to impact age-related CVDs. The ultimate goal is translating this knowledge to treat human CVDs [161,162]. Generally, there are two strategies for therapeutic targeting of noncoding RNAs: (1) restoring the function of noncoding RNAs that are insufficiently expressed; (2) blocking the actions of noncoding RNAs that are aberrantly overexpressed. 

Both these approaches are applicable to miRNAs. Indeed, since miRNAs have a small size, and are often cytoplasmic localized, their function can be restored with synthetic miRNA mimics, miRNAs vectors and small molecules. On the other hand, their function can be blocked by a variety of strategies such as LNA anti-miR, miR-sponges, and antagomiRs. 

For long noncoding RNAs, the overexpression and silencing approaches are in many aspects similar to those of coding mRNA. Unlike mature mRNAs, certain lncRNA are mostly nuclear and can be efficiently targeted by GapmeRs. 

## Figures and Tables

**Table 1:** LncRNA/miRNA/mRNA networks and ageing mechanisms involved in cardiovascular diseases.

Process	lncRNA	miRNA	Prediction Tool	miRNA Target (Direct/Indirect)	Pathology/Condition	Ref.
Autophagy	APF	miR-188-3p	RNAhybrid	ATG7 (direct)	-A/R in mouse CMs -I/R (mouse)	[71]
	AK088388	miR-30a	miRANDA TargetScan	Beclin-1 (direct) LC3-II (indirect)	-H/R in mouse CMs	[72]
	AK139328	miR-204-3p	nd	ATG proteins (indirect)	-Diabetic mouse -I/R (mouse)	[73]
	BACE1-AS	miR-29/miR-107/miR-124/miR-485/miR-761	miRANDA	BACE1 (direct)	-human DCM -HAOECs -mouse cardiomyocytes	[51,74,75]
	Galont	miR-338	RNAhybrid	ATG5 (direct)	A/R cardiomyocytes (mouse)	[76]
	GAS5	miR-26a	RNAhybrid	ATG proteins (indirect)	-ox-LDL stimulation of human ECs -plasma of AS patients	[77]
	TGFB2-OT1	miR-4459	Mirbase MicroInspector	ATG13 (direct)	-3BDO stimulation of human ECs	[78]
	UCA1	miR-128	nd	HSP70 (direct)	-I/R (mouse) -H/R in mouse CMs	[79]
Inflammation	GAS5	miR-26a	Starbase v2.0 LncBase Predicted v.2 tool	HMGB1	-PA treated mouse CMs	[80]
	GAS5	miR-221	nd	IL-1β, TNF-α, MMP-2, MMP-9 (indirect)	-Ox/LDL stimulation of THP-1 human cells -Human AS plaques	[81]
	H19	let-7	nd	Periostin	-Ox/LDL stimulation of human HUVEC	[82]
	HOTAIR	miR-34a	miRANDA	SIRT1 (direct)	-STZ mice -high glucose treated rat CMs	[83]
	MALAT-1	miR-155	TargetScan	SOCS1 (direct)	- ox-LDL stimulation of human HAOECs	[84]
	RNCR3	miR-185-5p	TargetScan	KLF-2 (direct)	-Human aortic lesions -Aorta from apoE^−/−^ mice - ox-LDL stimulation of human EC and VSMCs	[85]
	RP5-833A20.1	miR-382-5p	miRBase, PicTar, TargetScan, RNAhybrid	NFIA (direct)	-Ox/Ac-LDL stimulation of THP-1 human cells -Aorta from apoE^−/−^ mice	[86]
	TGFB2-OT1	miR-4459	MicroInspector	LARP/CERS1/ NAT8L/ ATG13 (direct)	-LPS/ox-LDL stimulation of human ECs	[87]
Senescence	GAS5	miR-223	miRWalk miRANDA TargetScan microT-CDS	NAMPT (direct)	-Human EPCs (late passages)	[88]
	H19	miR-29b-3p	nd	cIAP1 (direct)	-D-galactose/H-Post treatment of CMs -I/Post (mouse)	[89]
	LncRNA-ES3	miR-34c-5p	nd	BMF	-high glucose stimulated human aorta VSMCs	[90]
	MEG3	miR-128	nd	GIRDIN (direct)	-Coronary artery aged mice -HUVECs (late passages)	[91]

Abbreviations: A/R = Anoxia/reperfusion; AS = aortic stenosis; CMs = cardiomyocytes; DCM = dilated cardiomyopathy; ECs = endothelial cells; EPCs = endothelial precursor cells; HAOECs = human aortic endothelial cells; H-Post = hypoxia-postconditioning; H/R = hypoxia/reperfusion; HUVECs = human umbilical endothelial cells; I-Post = ischemia-postconditioning; I/R = Ischemia/reperfusion; LPS = lipopolysaccharide; ox-LDL= oxidized low density lipoprotein; PA = palmitic acid; STZ = streptozotocin; VSMCs = vascular smooth muscle cells; 3BDO = 3-benzyl-5-((2-nitrophenoxy) methyl)-dihydrofuran-2(3H)-one.
